# Supplementary material for: mdCATH: A Large-Scale MD Dataset for Data-Driven Computational Biophysics
Source: Sci Data. 2024 Nov 28;11:1299. doi: 10.1038/s41597-024-04140-z (PMC11604666; doi:10.1038/s41597-024-04140-z)
Supplement: Supplementary file 1 — Supplementary information [file 41597_2024_4140_MOESM1_ESM.pdf]

Supplementary Information

mdCATH: A Large-Scale MD Dataset for Data-Driven Computational Biophysics

Antonio Mirarchi Toni Giorgino Gianni De Fabritiis

October 24, 2024

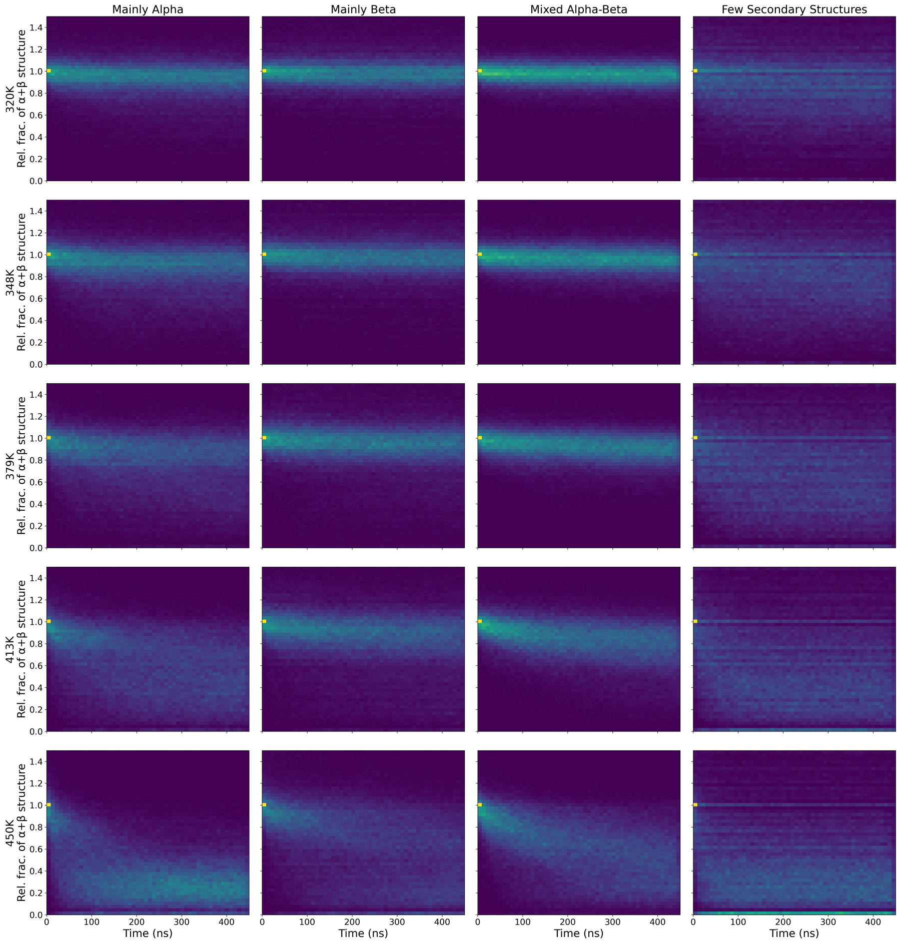

Figure S1: Stability of α/β structures in CATH protein superfamilies 1 to 4 at varying temperatures. The fraction (relative to $t = 0$) of secondary structure (y axis) is shown versus time (x axis) for the 4 main CATH protein classes. Data combine 200 domains, 50 for each superfamily, and all replicas into distribution profiles. β-dominated structures maintain stability up to 450 K, α-dominated structures show partial loss of secondary structure at 413 K and rapid deterioration at 450 K, and mixed α-β structures display intermediate stability. The *few secondary structures* class (fourth column) is more loosely defined and has lower stability. The class *special* is underrepresented and therefore not shown.
